# EFFECTIVENESS OF DUAL-LANGUAGE COGNITIVE INTERVENTION IN COGNITIVE HEALTHY AND DEMENTIA BILINGUAL OLDER ADULTS

**DOI:** 10.1093/geroni/igae098.3605

**Published:** 2024-12-31

**Authors:** W Quin Yow, Chi Shuen Ang, Xiaoqian Li

**Affiliations:** Singapore University of Technology & Design, Singapore, Singapore; Singapore University of Technology & Design, Singapore, Singapore; Singapore University of Technology & Design, Singapore, Singapore

## Abstract

There is an increasing interest in using touch-screen devices to conduct cognitive training and collect measurements of cognitive performance. Although research with technology-based intervention has shown benefits in improving cognitive performance with older adults, overall results are mixed (Ge et al, 2018). This study aims to investigate whether our elder-friendly, multi-modal touch-screen, computerized dual-language intervention program would help to improve cognitive skills in cognitive healthy and dementia bilingual older adults in a multilingual society. Fifty-eight older adults, 26 with dementia (Mage 82.77 ± 7.45) and 32 healthy older adults (Mage 72.44 ± 8.42), completed 24 sessions of gameplay over 8-12 weeks. Each session took about 30-45 minutes to complete, and consisted of two cognitive tasks: Object Categorization (OC), and Utility of things (UoT). Participants’ performance was assessed by reaction time (RT) and number of prompts used to reduce task difficulty. Using a two-way ANOVA, we compared the performance of the first 4 and last 4 sessions of the two groups for both tasks. By the last 4 sessions, there were significant improvements in performance of the dementia group (i.e., faster RT and fewer prompts for OC, and fewer prompts for UoT; post-hoc ps<.002). Performance in the cognitively-healthy group remained stable (ps>.11), and they consistenly outperformed the dementia group across the intervention (ps<.001). These improvements suggest that our cognitive intervention program could be effective for older adults with dementia. In sum, bilingual elder-friendly touch-screen platforms can be a novel yet effective method to study cognitive interventions in populations of older multilinguals.

